# Characterization of Quantitative Trait Loci for Germination and Coleoptile Length under Low-Temperature Condition Using Introgression Lines Derived from an Interspecific Cross in Rice

**DOI:** 10.3390/genes11101200

**Published:** 2020-10-15

**Authors:** Mirjalol Akhtamov, Cheryl Adeva, Kyu-Chan Shim, Hyun-Sook Lee, Sun Ha Kim, Yun-A Jeon, Ngoc Ha Luong, Ju-Won Kang, Ji-Yoon Lee, Sang-Nag Ahn

**Affiliations:** 1 Department of Agronomy, Chungnam National University, Daejeon 34134, Korea; miramax90501@gmail.com (M.A.); ccadeva_758@yahoo.com (C.A.); zktnrl@naver.com (K.-C.S.); leehs0107@gmail.com (H.-S.L.); sunha82@cnu.ac.kr (S.H.K.); jya0911@cnu.ac.kr (Y.-AJ.); luongngocha.biotech@gmail.com (N.H.L.); 2 Department of Southern Area Crop Science, National Institute of Crop Science, RDA, Miryang 50424, Korea; kangjw81@korea.kr (J.-W.K.); minitia@korea.kr (J.-Y.L.)

**Keywords:** low-temperature germinability, rice, interspecific cross, QTL, interaction

## Abstract

Previously, five putative quantitative trait loci (QTLs) for low-temperature germination (LTG) have been detected using 96 BC_3_F_8_ lines derived from an interspecific cross between the Korean *japonica* cultivar “Hwaseong” and *Oryza rufipogon*. In the present study, two introgression lines, CR1517 and CR1518, were used as parents to detect additional QTLs and analyze interactions among QTLs for LTG. The F_2_ population (154 plants) along with parental lines, Hwaseong and *O. rufipogon*, were evaluated for LTG and coleoptile length under low-temperature conditions (13 °C). Among five QTLs for LTG, two major QTLs, *qLTG1* and *qLTG3*, were consistently detected at 6 and 7 days after incubation. Three minor QTLs were detected on chromosomes 8 and 10. Two QTLs, *qLTG10.1* and *qLTG10.2*, showing linkage on chromosome 10, exerted opposite effects with the Hwaseong allele at *qLTG10.2* and the *O. rufipogon* allele at *qLTG10.1* respectively, in turn, increasing LTG. Interactions among QTLs were not significant, implying that the QTLs act in an additive manner. Near-isogenic line plants with the combination of favorable alleles from *O. rufipogon* and Hwaseong exhibited higher LTG than two introgression lines. With regard to coleoptile length, three QTLs observed on chromosomes 1, 3, and 8 were colocalized with QTLs for LTG, suggesting the pleiotropy of the single gene at each locus. According to the results, the introgression of favorable *O. rufipogon* alleles could hasten the development of rice with high LTG and high coleoptile elongation in *japonica* cultivars.

## 1. Introduction

Rice is one of the most important crops, feeding more than a third of the global population. Global rice demand is projected to rise from 723 million tons in 2015 to 763 million tons by 2020 and is expected to increase further to 852 million tons by 2035 [1]. To feed the growing population, rice production needs to be improved and stabilized, globally and regionally. Improving potential yield and incorporation of biotic and abiotic stress tolerance mechanisms would facilitate the achievement of such goals. Wild relatives of rice are rich sources of desirable genes, not only with regard to yield but also with regard to disease resistance, stress tolerance, and other traits [2,3]. Exploring wild and exotic rice germplasm for desirable genes and transferring them into cultivars through crossing and marker-assisted selection (MAS) has been demonstrated to be feasible approaches of increasing rice yield and augmenting stress tolerance [4].

Rice is cultivated in tropical, subtropical, and temperate regions of the globe. Low-temperature is one of the abiotic stress factors that lead to growth retardation and yield loss in rice cultivated at high latitudes and in the northern regions of the globe [5]. Throughout the rice cultivation period, decreasing temperatures to levels lower than the optimum could influence rice development significantly and can result in severe yield loss [6]. During the seed development period, various factors, such as environment and genetic background, influence low-temperature germination [7]. In rice cultivating regions, in areas where rice is irrigated with cold water, weak seedling growth and slow growth reduce yield significantly [8,9,10,11,12,13].

Along with rapid low-temperature germination (LTG), vigorous coleoptile growth is essential in direct seeding rice, when rice seeds are sown in flooded paddy fields and watered with cold irrigation water [14]. The rice coleoptile is a small and ephemeral organ that emerges first in imbibed rice seeds [15].

To determine the genetic basis of low-temperature germinability and coleoptile elongation in rice, numerous independent studies have been conducted using biparental populations and genome-wide association studies [6,8,9,12,16,17,18,19,20,21]. A total of 11 QTLs have been associated with low-temperature germination ability in the population derived from a cross between *indica* (N22) and *japonica* (USSR5) rice [12]. Three QTLs associated with low-temperature germination have been detected, and among them, *qLTG-9* was fine mapped to a 72.3-kbp region on chromosome 9 [22]. In addition, Lee et al. (2015) detected two colocalized QTLs for LTG and coleoptile length under a low-temperature condition (13 °C) [6]. Fukuda et al. (2015) also identified two QTLs associated with coleoptile length under 16 °C conditions [23]. Using the genome-wide association (GWAS) method, two QTLs associated with germination under cold tolerance, *qCTGERM8-1* and *qCTGERM8-2*, were identified in 421 accessions from a rice diversity panel (RDP1) [24]. In addition, Fujino et al. (2015) detected *qLTG8* on the RM5647 marker locus in 63 Japanese landraces [18]. Li et al. (2019) reported that Chinese Dongxiang wild rice (*O. rufipogon* Griff.) alleles at all five QTLs resulted in delayed germination rates, and QTL pyramiding line DX71 led to rapid germination and vigorous seedling survival under low-temperature conditions (15 °C and 8 °C) [25]. RNAseq analysis was conducted with two *indica* rice genotypes under cold treatment and detected 1361 differentially expressed transcripts [26].

Such studies indicate that low-temperature germination QTLs are distributed widely throughout the rice genome. However, the stability of such putative QTLs has not been tested in near-isogenic backgrounds, and studies analyzing interactions among QTLs are limited. Characterization of the QTLs underlying LTG and their interactions could provide more insights into the mechanisms of low-temperature germinability in rice and would be potentially more useful for pyramiding QTLs aimed at improving LTG in rice. 

In our previous study, five QTLs for low-temperature germinability, *qLTG1*, *qLTG3*, *qLTG4*, *qLTG10*, and *qLTG11* were mapped using a BC_4_F_8_ population including 96 introgression lines (ILs) derived from an interspecific cross between a Korean elite line Hwaseong and *O. rufipogon* [27]. Recently, *qLTG1* was fine mapped using introgression lines and the new *qLTG3-1* allele of the *O. rufipogon* was identified [28,29]. In the present study, we used two introgression lines, CR1517 and CR1518, which consistently exhibit higher LTG than Hwaseong due to *O. rufipogon* introgression segments in the Hwaseong genetic background, as parents. Using 154 F_2_ plants derived from a cross between CR1517 and CR1518, we aimed to: (1) identify and characterize QTLs for low-temperature germinability and coleoptile length, (2) analyze interactions among LTG QTLs, and (3) develop QTL pyramiding lines with enhanced LTG and coleoptile length.

## 2. Materials and Methods

### 2.1. Plant Materials

**Figure 1:**
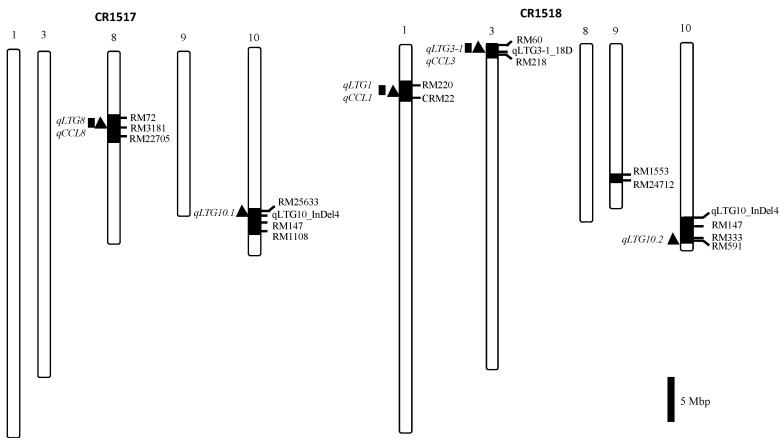
Graphical genotypes of two parental lines (CR1517 and CR1518) with the locations of five and three QTLs for low-temperature (*qLTG* in filled triangle) and coleoptile length (*qCCL* in filled box), respectively, on the left of the chromosomes. Black and white bars represent *O. rufipogon* and Hwaseong chromosome segments, respectively.

Two introgression lines, CR1517 and CR1518, which were derived from crossing between Hwaseong and *O. rufipogon*, were used as parents. We selected the two parental lines from an interspecific cross population, BC_4_F_8_ [27]. CR1517 has *O. rufipogon* chromosomal segments on chromosomes 8 and 10, and CR1518 has *O. rufipogon* segments on chromosomes 1, 3, 9, and 10 (Figure 1). Two introgression lines were crossed to produce F_1_ seeds. During the winter season of 2018–2019, F_1_ seeds were sown in a greenhouse to produce F_2_ seeds. In the middle of April 2019, F_2_ seeds were sown in a greenhouse and 30-day-old seedlings were transplanted into the experimental paddy field. The F_2_ population (154 plants) was grown in a field belonging to Chungnam National University, Daejeon, South Korea. The plants were used for further phenotypic and genotypic analyses. To verify minor QTLs, two F_2_ plants (CR8017-4 and CR8020-6) were selected based on the genotypes to verify *qLTG10.1* and *qLTG10.2*, respectively. CR8017-4 is heterozygous at *qLTG10.1* and *qLTG8*, while *O. rufipogon* homozygous for *qLTG3*, and Hwaseong homozygous for *qLTG1* and *qLTG10.2*. CR8020-6 is heterozygous at *qLTG10.2*, while *O. rufipogon* homozygous for *qLTG1* and Hwaseong homozygous for *qLTG3* and *qLTG10.1*. The F_3_ plants were grown in the 2019/2020 winter season and dormancy was broken for seeds harvested from each F_3_ plant.

### 2.2. Evaluation of LTG and Coleoptile Length

Seeds from each F_2_ plant were harvested five weeks after heading and dried in a greenhouse for two weeks. To break dormancy, seeds were stored in a dry oven at 50 °C for 72 h. For normal temperature and low-temperature germination tests, 20 and 30 seeds were used with three replicates, respectively. Healthy and normal seeds were carefully selected and used in the germination test. The seeds from each plant were placed into 9-cm Petri dishes onto one layer of filter paper and 5 mL of distilled water added. Dormancy breakage was checked at the normal germination temperature (30 °C) for four days. The low-temperature germination test was conducted at 13 °C under the dark condition for eight days. Germinated seeds of each plant were counted daily and defined as germination rate. The germination rate (%) was calculated as follows: Germination rate (%) for a given day = (number of germinated seeds until the given day/total number of seeds) × 100. The germination tests were repeated two times under similar conditions. The seeds were considered germinated when coleoptile emerged from the seed (Figure S1).

Coleoptile length measurement was conducted based on previous studies [6,14]. F_3_ seeds of F_2_ plants were used to measure coleoptile length. Twenty normal and healthy seeds per plant were placed on one layer of filter paper in 9-cm Petri dishes and 10 mL of distilled water was poured into each Petri dish. The measurements were performed with two replicates using parental lines under normal (30 °C) and low-temperature (13 °C) conditions. In the F_3_ generation, coleoptile length was measured under low-temperature conditions (13 °C) with one replicate. Distilled water was renewed daily and maintained at 30 °C and 13 °C conditions for the normal and low-temperature conditions, respectively. Coleoptile length was measured using a ruler. In the normal temperature treatments, measurements were carried out from 1 to 4 days after incubation (DAI) and, in the low-temperature treatments, measurements were carried out from 5 to 10 DAI. For the normal and low-temperature conditions, coleoptile lengths of the grains were measured at room temperature (26 °C) and at seed storage temperature (10 °C) conditions, respectively, and mean lengths of the coleoptile were used in further analyses.

### 2.3. DNA Extraction and Marker Analysis

Booting stage plant leaves were gathered for use in extracting genomic DNA, and extraction was performed based on the method of Causse et al. (1994) with some minor modifications [30]. The Cetyl trimethylammonium bromide (CTAB) method was used to extract plant DNA. Leaf fragments from each plant were placed in a 2.0-mL microcentrifuge tube with aluminum beads (4 mm) and 500 µL of CTAB extraction buffer added. The tubes were incubated at 55 °C in a dry oven in CTAB buffer (2% CTAB, 100 mM pH 8.0 Tris-HCI, 20 mM EDTA, 1.4 M NaCl, 0.2% β-mercaptoethanol) for 30 min right after grinding. An equal volume of chloroform was added and the solution was mixed well. The tubes were centrifuged at 4 °C for 15 min at 13,000 rpm. The 200-µL upper supernatant layer was transferred into a new 1.5-mL tube and mixed with 2/3 volume of isopropanol, and after gently mixing, incubated at 4 °C for 15 min at 12,000 rpm. The supernatant was discarded very carefully, and the DNA pellets were washed with 70% ethanol once. After washing the DNA pellets, they were kept at room temperature to dry out the rest of ethanol and then diluted in 100 μL TE buffer (10 mM Tris pH 8, 1 mM EDTA). The concentration of the DNA was checked with a NanoDrop^TM^ 2000 spectrophotometer (Thermo Scientific Inc., Wilmington, DE, USA).

A 15-µL of PCR reaction mixture containing 5 µL DNA template (2~5 ng/µL), 1 µL of forward and reverse primer (10 pmol each), 1.5 µL of 10× PCR buffer (10 mM Tris-HCl pH 8.3, 50 mM KCl, 1.5 mM MgCl_2_, 0.1% Gelatin), 1 µL of dNTP (2.5 mM each), 0.1 µL of *Taq* polymerase (5 unit/µL), and 6.4 µL triple distilled water was used. The PCR was performed as described in Panaud et al. (1996) with minor modifications: 94 °C for 5 min, followed by 33–35 cycles of 94 °C for 30–40 s, 52–60 °C (based on annealing temperature of each marker) for 30 s, and 72 °C for 30 s, and a final extension at 5 min at 72 °C [31]. Separation of PCR products was conducted on 3% of metaphor agarose gel stained with Staining Safe Nucleic Acid Gel Stain (RBC, Taiwan). A total of 18 markers, including 15 Simple Sequence Repeat (SSR) and 3 Insertion-Deletion (InDel) markers were used to map QTLs (Table S1). Three InDel markers (qLTG3-1_18D, qLTG10_InDel3, and qLTG10_InDel4) and one SSR marker (CRM22) were designed and used for mapping [27,28].

### 2.4. Statistical Analysis and QTL Analysis

QTL analyses were conducted in Minitab 16.2.4 (Minitab Inc., State College, PA, USA) using one-way Analysis of Variance and Tukey’s test for mean comparisons. QTLs were detected using single-marker analysis, and the QTL was declared when the association between phenotype and genotype of markers was significant at *p* < 0.05. Gene actions were calculated by the following formula; additive effect (*a*) = (mean germination rate of *O. rufipogon* homozygote − mean germination rate of Hwaseong homozygote)/2; dominant effect (*d*) = mean germination rate of heterozygote − (mean germination rate of *O. rufipogon* homozygote + mean germination rate of Hwaseong homozygote)/2; degree of dominance = *d*/*a*. To analyze the interaction between QTLs, a general multiple regression model with two QTLs as independent variables and interaction term was employed in Minitab 16.2.4. Nomenclature of the QTLs has been described previously [32].

## 3. Results

### 3.1. Low-Temperature Germination Rate of Parental Lines and F_2_ Population

Germination rates of four parental lines (Hwaseong, *O. rufipogon*, CR1517, and CR1518) were compared under control conditions (30 °C). The seeds of *O. rufipogon* began germinating at 1 DAI, with a 47% germination rate. At 4 DAI, *O. rufipogon* germination rate reached 100%. Hwaseong, CR1517, and CR1518 began germinating 2 DAI. Subsequently, the germination rate of the lines achieved nearly 100% at 4 DAI. *O. rufipogon* exhibited a significantly higher germination rate than those of other lines from 1 to 2 DAI. There were no significant differences in germination rate among the parental lines from 3 to 4 DAI under the control temperature (Figure S2). However, there were differences in the germination rate between 4 lines under the low-temperature condition (13 °C). *O. rufipogon* began germinating at 3 DAI and attained 100% germination rate at 7 DAI. In contrast, Hwaseong began germinating 4 DAI, and achieved 80% germination 8 DAI. Two introgression parental lines, CR1517 and CR1518, began germinating at 3 DAI, and the lines attained 85% and 94% germination rates, respectively, 7 DAI. They exhibited much more vigorous germinability than that of Hwaseong and lower than that of *O. rufipogon* (Figure S2). Germination rates in F_2_ plants ranged from 0 to 100% during the incubation period for 1–8 DAI (Table 1). The results revealed that *O*. *rufipogon* segments increased LTG in the Hwaseong background.

### 3.2. Mapping QTLs for Low-Temperature Germinability

**Figure 2:**
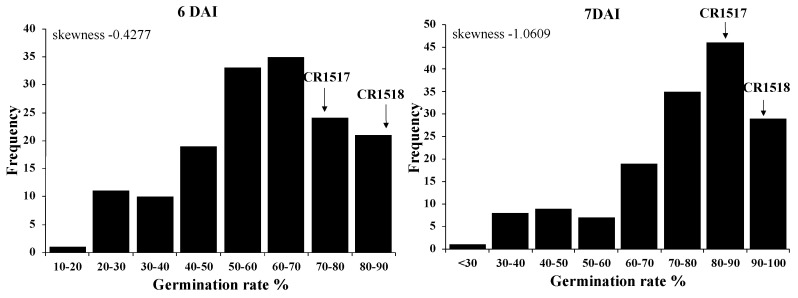
Frequency distribution of germination rate in 154 F_2_ plants at 6 and 7 days after incubation (DAI) at 13 °C. Germination rate was measured at 4–8 (DAI). Arrows indicate mean germination rate of CR1517 and CR1518 lines.

We used low-temperature germination rate of the F_2_ population at 6 and 7 DAI for the QTL analysis because we observed larger differences among the parental lines (CR1517 and CR1518) at the periods. The F_2_ population exhibited nearly normal distribution of the LTG rate at 6 DAI (skewness = −0.4277) and was skewed to the left at 7 DAI (skewness = −1.0609) (Figure 2). Using QTL-linked SSR markers and one InDel marker, we consistently detected *qLTG1* and *qLTG3* 6 and 7 DAI, respectively. *qLTG1* was detected between RM220 and CRM22 markers, while *qLTG3* was detected on qLTG3-1_18D marker on chromosomes 1 and 3, respectively. The *qLTG1* explained 16.0% and 12.0% phenotypic variation 6 and 7 DAI, respectively, while *qLTG3* explained 23.8% and 22.1% phenotypic variation 6 and 7 DAI, respectively. Based on gene action analysis 6 DAI, the additive effect (*a*) and dominant effect (*d*) of *O. rufipogon* allele on *qLTG1* locus (RM220–CRM22) were 10.0 and 0.2, respectively. *O. rufipogon* allele of *qLTG1* had 7.7 and 2.1% of the additive and dominant effects, respectively, 7 DAI. In the *qLTG3* locus (qLTG3-1_18D), *O. rufipogon* allele accounted for 9.6 and 8.1% of the additive effects 6 and 7 DAI, respectively. The dominant effect was 9.7% on both days. Degrees of dominance were 1.0 and 1.2 at 6 and 7 DAI, respectively (Table 2). In addition, we detected two minor QTLs on chromosome 8 and 10. No QTLs were detected between markers RM72 and RM22705 (*p* = 0.086) on chromosome 8 at 6 DAI. *qLTG8* was detected when the significant difference had a *p* value of 0.014 at 7 DAI. The phenotypic variation explained by *qLTG8* was 5.45%. In addition, we identified other QTLs on chromosome 10. The QTLs were detected on the most distal end of the short arm of chromosome 10 by RM25633 and RM333-RM591, respectively. The phenotypic variation explained by *qLTG10.1* was 4.6% at 7 DAI. *qLTG10.2* explained 4.2 and 6.6% of the phenotypic variation at 6 and 7 DAI, respectively. In addition, additive and dominant effects of *O. rufipogon* alleles at the *qLTG8* locus (RM72-RM22705) were 4.9 and 2.4% at 7 DAI, respectively. *qLTG10.1* had 2.9 and −1.9% additive and dominant effects, respectively, at 7 DAI. The additive effects of *qLTG10.2* were −4.2 and −5.3% at 6 and 7 DAI, and the dominant effects of the QTL were 5.1 and 5.5% at 6 and 7 DAI, respectively. Although two QTLs, *qLTG10.1* and *qLTG10.2*, were linked on the short arm of chromosome 10, the effect for LTG of *O. rufipogon* allele was different. *O. rufipogon* introgression had a −0.6 degree of dominance at the *qLTG10.1* locus at 7 DAI, while −1.2 and −1.0 degrees of dominance were observed at *qLTG10.2* at 6 and 7 DAI, respectively (Table 2). The gene actions of *O. rufipogon* are different in the five QTLs. The *O. rufipogon* allele is partially dominant at *qLTG8* and *qLTG10.2*, partially recessive at *qLTG10.1*, completely dominant at *qLTG3*, and completely recessive at *qLTG1* with regard to regulating LTG (Table 2). 

### 3.3. Interaction between LTG QTLs

Analysis of interactions among QTLs was carried out using the average low-temperature germination rate of nine genotypic classes at 7 DAI in the F_2_ population. Based on the general regression model, no significant interaction was detected between five QTLs. Although two QTL, *qLTG3* and *qLTG8* (*p* = 0.145) showed relatively high QTL interaction at 7 DAI, no significant QTL interaction at *p* < 0.05 was observed. In addition, the presence of *O. rufipogon* allele at the *qLTG8* locus increased LTG in three genotype classes at the *qLTG1* and *qLTG3* loci (Figure S3A,D). The highest LTG scores were observed in the genotype classes with *O. rufipogon* homozygous at *qLTG10.1* and Hwaseong homozygous at *qLTG10.2*. The results suggest that the *O. rufipogon* alleles at *qLTG1*, *qLTG3*, *qLTG8*, and *qLTG10.1* and the Hwaseong allele at *qLTG10.2* increased LTG, and QTLs act in an additive manner to regulate LTG. 

### 3.4. QTL Pyramiding Line for LTG

We compared the mean low-temperature germination rates of the F_2_ QTL-pyramiding lines (QTL-PL) with those of the parental lines (*O. rufipogon*, Hwaseong, CR1517, and CR1518) (Table S2). QTL-PL represents *O. rufipogon* alleles at four QTLs, *qLTG1*, *qLTG3*, *qLTG8*, and *qLTG10.1*, and Hwaseong allele at *qLTG10.2* locus. QTL-PL had higher germination rates than CR1517, CR1518, and Hwaseong, and lower germination rates than *O. rufipogon* at 6 and 7 DAI under the 13 °C condition. Although an 87.3% germination rate was observed in QTL-PL, this rate was significantly higher than that of CR1517 but not different from CR1518 at 6 DAI. The results indicate that pyramiding the QTLs with the combination of *O. rufipogon* alleles at *qLTG1*, *qLTG3*, and *qLTG8*, *qLTG10.1* and Hwaseong allele at *qLTG10.2* led to robust germinability under low-temperature conditions (13 °C). The lines could be useful material for developing rice varieties with enhanced LTG capacity (Table S2).

### 3.5. Verification of LTG QTL Using F_3_ Population

**Figure 3:**
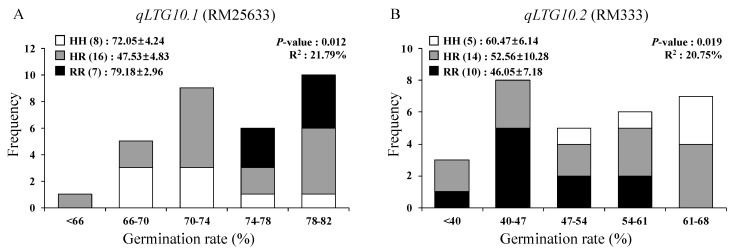
Frequency distribution of LTG at 7 days after incubation in the two F_3_ population. Plants in each population are segregating in (**A**) RM25633 for *qLTG10.1* and (**B**) RM333 for *qLTG10.2* in the different genetic background. The numbers in parenthesis indicate plants number followed by mean germination rate ± standard deviation of genotype group. *p*-value and R^2^ were determined by one-way ANOVA. HH: Hwaseong homozygoutes, RR: *O. rufipogon* homozygotes, HR: heterozygotes.

Two F_2_ plants (CR8017-4 and CR8020-6) were selected based on their genotypes to verify *qLTG10.1* and *qLTG10.2*, respectively. CR8017-4 is heterozygous at *qLTG10.1* and *qLTG8*, while *O. rufipogon* homozygous for *qLTG3* and Hwaseong homozygous for *qLTG1* and *qLTG10.2*. CR8020-6 is heterozygous at *qLTG10.2*, while *O. rufipogon* homozygous for *qLTG1* and Hwaseong homozygous for *qLTG3* and *qLTG10.1*. The F_3_ plants were grown in the 2019/2020 winter season and dormancy was broken for seeds harvested from each F_3_ plant. We used low-temperature germination rates of two F_3_ populations at 6 and 7 DAI for the QTL analysis. QTL analysis indicated that RM25633 explained 21.79% of the phenotypic variance in the CR8017-4 population, confirming the presence of *qLTG10.1* (Figure 3A). However, we failed to detect the effect of *qLTG8* (*p* = 0.21). Additional experiments using larger populations are needed to verify the effect of *qLTG8*. QTL analysis indicated that *qLTG10.2* explained 20.75% of the phenotypic variance in the CR8020-6 population (Figure 3B). The Hwaseong allele at *qLTG10.2* contributed to an increase in LTG. The results indicate that two linked QTLs (*qLTG10.1* and *qLTG10.2*) on chromosome 10 are associated with LTG. Because *O. rufipogon* allele at *qLTG10.1* and Hwaseong at *qLTG10.2* contributed to higher LTG, the *O. rufipogon qLTG10.1* could be selectively introgressed into *japonica* rice using MAS. Two QTLs, *qLTG10.1* and *qLTG10.2*, explained 21.79% and 20.75% of the phenotypic variance, respectively, in the near-isogenic background. The low contribution of the QTL is partly due to the small population size and other environmental factors. The F_3_ plants were grown in the 2019/2020 winter season. We recorded the heading date of each plant and harvested them 30 days after heading. However, some late-flowering F_3_ plants failed to set adequate seeds to be tested for LTG, leading to the small population size and uneven distribution of three genotypic classes in the F_3_ population. 

### 3.6. Coleoptile Length in Parental Lines and F_2_ Population

*O. rufipogon* showed significantly higher coleoptile length during 2–3 DAI at optimal condition than Hwaseong, CR1517, and CR1518, while no significant difference was observed in coleoptile length among Hwaseong, CR1517, and CR1518 at 30 °C (Figure S4A). At 1 DAI, mean coleoptile lengths were 0.6 mm and 0.3 mm in *O. rufipogon* and Hwaseong, respectively, while CR1517 and CR1518 were 0.4 mm in length on average. Coleoptile length varied from 12.4 mm to 14.6 mm at 4 DAI among parental lines (Figure S4A).

Coleoptile length varied markedly among parental lines under the 13 °C condition (Figure S4B). The mean length of the coleoptile in *O. rufipogon* was longer than those of Hwaseong, CR1517, and CR1518. The average coleoptile length in *O. rufipogon* at 5 DAI was 1.5 mm, while it reached 9 mm at 10 DAI. In Hwaseong, it was close to zero at 5 DAI and attained 4 mm at 10 DAI. While the average coleoptile length in CR1518 was slightly greater than that in Hwaseong at 5 to 9 DAI, the difference was much greater at 10 DAI. CR1517 had a longer coleoptile than CR1518 and Hwaseong, and a shorter coleoptile than *O. rufipogon*. The differences in coleoptile length between parental lines were more significant at 9 and 10 DAI than at other scoring dates (Figure S4, Table 3).

**Figure 4:**
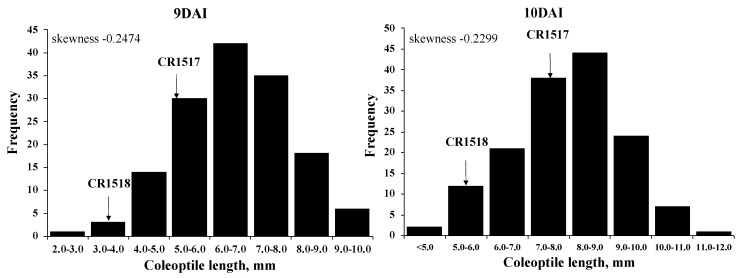
Frequency distribution of coleoptile length in 149 F_2_ plants at 13 °C at 9 and 10 DAI. Arrows indicate mean coleoptile length of CR1517 and CR1518 lines.

In the F_2_ population, the coleoptile length varied from 0 to 12 mm from 5 to 10 DAI. The mean coleoptile lengths of 149 F_2_ plants were 0.4, 0.7, 1.2, and 3.2 mm at 5, 6, 7, and 8 DAI, respectively. At 9 DAI, the average coleoptile length was 6.6 mm, and it reached 7.9 mm at 10 DAI (Table 4, Figure 4). The F_2_ population exhibited nearly normally distributed coleoptile elongation at 9 and 10 DAI (Figure 4).

### 3.7. QTLs for Coleoptile Length

QTL analysis of coleoptile length was carried out for two scoring dates (9 and 10 DAI) considering that the two parental lines had the greatest difference in coleoptile length (Table 3). A total of three QTLs associated with coleoptile length were detected and they were colocalized with the LTG QTLs (*qLTG1*, *qLTG3*, and *qLTG8*). At 9 DAI, *qCCL3* (qLTG3-1_18D) and *qCCL8* (RM22689-RM22705) were identified and they explained 12.0 and 6.4% of the phenotypic variance, respectively. The *qCCL1* on chromosome 1 between markers RM220 and CRM22 was not significant (*p* = 0.059) at 9 DAI (data not shown).

At 10 DAI, *qCCL1*, *qCCL3,* and *qCCL8* were detected (Table 4). The QTLs explained 6.4%, 9.2%, and 7.1% of the total phenotypic variance, respectively. No QTL was detected on the other *O. rufipogon* introgression segments on chromosomes 9 and 10 at 9 and 10 DAI. *O. rufipogon* alleles are partially recessive at *qCCL1* and *qCCL8* loci, and dominant at the *qCCL3* locus similar to the case in LTG (Table 2 and Table 4). *qLTG8* in this study was colocalized with the *qCCL8* for coleoptile length under cold stress in the study [6] and the allelic relationship between two QTLs remains to be clarified.

## 4. Discussion

Rice is one of the most important staple food crops globally, being consumed by approximately 50% of the global population [33]. Low-temperature germinability (LTG) is one of the major factors influencing stable crop establishment in the direct seeding method of rice cultivation in tropical and subtropical regions of the world. Along with rapid low-temperature germination, vigorous coleoptile growth is essential in the direct-seeding method of rice when rice seeds are sown in flooded paddy fields and watered with cold irrigation water [14]. Rapid coleoptile elongation after germination is necessary to improve seedling establishment rate [6].

In the present study, we analyzed LTG and coleoptile length in rice cultivated under 13 °C conditions. Using the F_2_ population derived from a cross between two introgression lines, CR1517 and CR1518, we detected a total of five and three QTLs for LTG and coleoptile length, respectively, over two scoring dates. QTLs associated with LTG were detected on chromosomes 1, 3, 8, and 10. Among them, two major QTLs, *qLTG1* and *qLTG3*, were detected on chromosomes 1 and 3, respectively. One minor QTL for coleoptile elongation, *qLTG8*, together with *qCCL8*, was detected between RM72 and RM22705 markers on chromosome 8. We failed to detect the QTL using 96 introgression lines in a previous study [27], which could be due to a masking effect of the major QTLs (*qLTG1* and *qLTG3*) and the genetic structure of the population [27]. Several studies have reported the presence of QTLs for LTG and coleoptile elongation on chromosome 8 [5,6,18,34]. For example, Najeeb et al. (2020) detected an LTG QTL, *qLTG(III)8* (497SNP_8_8509144), which is located near *qLTG8* in the present study [34]. The results suggest that the region on chromosome 8 participates in the regulation of germination and coleoptile elongation under low-temperature conditions. Identification of such genes from diverse varieties could enhance our understanding of the roles of the region in germination activities.

Low-temperature germination QTLs are distributed widely throughout the rice genome, including on chromosome 10 [12,17]. We detected two linked QTLs, *qLTG10.1* and *qLTG10.2* for LTG, with an LTG-increasing allele originating from *O. rufipogon* at *qLTG10.1* and Hwaseong at *qLTG10.2*. The results were confirmed in the near-isogenic background in the F_3_ population. Notably, the locations of *qLTG10.1* and *qLTG10.2* (20.0~22.9 Mb region) are similar to that of *qGR-10* for low-temperature germination ability detected between C809 and C797 on chromosome 10 (21.0~22.0 Mb region) in a study by Ji et al. (2009) [17]. They mapped *qGR10* using a recombinant inbred line population derived from a cross between Asominori and IR24. At *qGR-10*, the IR24 allele increased germination rate at two scoring dates (8 and 9 DAI), whereas the Asominori allele increased germination rate at later scoring dates (10 and 14 DAI). Similar results have been observed, where the beneficial alleles for LTG originate from two parents, WTR-1 and Haoannong, at two linked loci, *qLTG(I1)*_11_ and *qLTG(III)*_11_, on chromosome 11 [34]. Li et al. (2019) also observed that Dongxiang wild rice (*O. rufipogon* Griff.) introgressions at five detected QTLs in 94 BC_1_F_7_ population delayed germination rates under 15 °C conditions in the background of *indica* variety, Xieqingzao. Among them, two QTLs, *qLTG10-1* and *qLTG10-2*, were identified on chromosome 10 [25]. The results indicate that two linked QTLs potentially act in opposite directions in such QTL regions. Such genetic linkage is common in rice [35]. A linkage between two desirable genes would be advantageous in the selection of improved lines. For example, the tight linkage of two QTLs (*qSPP5* for spikelet no. and *qTGW5* for grain weight) could be valuable for improving rice yield [36]. However, linkage between desirable and undesirable genes is complex in terms of its application in rice breeding [37]. Since two LTG QTLs, *qLTG10.1* and *qLTG10.2*, acting in opposite directions are linked and have minor effects, selection of the high LTG lines with *qLTG10.1* from *O. rufipogon* and *qLTG10.2* could be accomplished using DNA markers. In a previous study, we detected *qLTG10.1* and *not qLTG10.2*, possibly due to the buffering effect of two QTLs and interactions among QTLs in the population [27]. Overall, it appears that the region carries gene(s) with a strong effect on germination performance and represent additional genetic targets for MAS directed development of rice varieties with improved LTG.

The interactions among the four QTLs, *qLTG1*, *qLTG3*, *qLTG8*, *qLTG10.1*, and *qLTG10.2* were examined using general regression models. The plants that harbor the *O. rufipogon* alleles at *qLTG1*, *qLTG3*, *qLTG8*, and *qLTG10.1* exhibited the highest germination rates at 13 °C in the nine groups, and the five QTLs cumulatively explained 42.0% of the phenotypic variance in LTG. The results imply that five QTLs control the LTG in an additive manner. Pyramiding the four QTLs from the *O. rufipogon* into cultivated rice with *qLTG10.2* would facilitate breeding programs aimed at enhancing LTG for direct-seeding production systems. It is also notable that the plants with four *O. rufipogon* alleles at *qLTG1*, *qLTG3*, *qLTG8*, and *qLTG10.1* exhibited lower LTG than *O. rufipogon*, the donor parent at 5–7 DAI, indicating the presence of additional QTLs for LTG in *O. rufipogon*. Further experiments are underway to detect and characterize such unknown QTLs in *O. rufipogon*.

Also, three QTLs for coleoptile length detected on chromosomes 1, 3, and 8 shared their locations with three LTG QTLs, *qLTG1*, *qLTG3*, and *qLTG8,* respectively, and the *O. rufipogon* alleles at all three loci increased the coleoptile length, suggesting a pleiotropy of a single QTL at each locus.

## 5. Conclusions

In the present study, we performed QTL analysis and identified QTLs for LTG and coleoptile length in the F_2_ population. Among the five QTLs for LTG, two major QTLs, *qLTG1* and *qLTG3*, and three minor QTLs were detected on chromosomes 8 and 10. Fine mapping revealed that two QTLs, *qLTG10.1* and *qLTG10.2*, were linked on chromosome 10 and exerted opposite effects with the Hwaseong allele at *qLTG10.2* and the *O. rufipogon* at *qLTG10.1*, respectively, in turn, increasing LTG. Because two LTG QTLs, *qLTG10.1* and *qLTG10.2*, which act in opposite directions are linked, the DNA markers could improve the selection efficiency of the high LTG lines with *qLTG10.1* from *O. rufipogon* and *qLTG10.2* from Hwaseong. Interactions among QTLs were not significant, implying that the QTLs act in an additive manner. NIL plants with combinations of favorable alleles from *O. rufipogon* and Hwaseong exhibited the highest LTG among all groups, supporting the absence of interactions. With regard to coleoptile length, three QTLs observed on chromosomes 1, 3, and 8 were colocalized with QTLs for LTG, suggesting the pleiotropy of the single gene at each locus. According to the results, the introgression of favorable *O. rufipogon* alleles should hasten the breeding of high LTG and coleoptile elongation in *japonica* cultivars.

## Figures and Tables

**Table 1:** Mean germination rates in *O. rufipogon*, Hwaseong, two introgression lines, and F_2_ population.

Germination Rate (%)						
Trait ^(x)^	Lines (*n* = 5 ^(z)^)				F_2_ Population	
	*O. rufipogon*	Hwaseong	CR1517	CR1518	Mean	Range
4 DAI	60.0 a ^(y)^	3.3 c	30.4 b	34.8 b	15.6 ± 10.1	1.1–48.9
5 DAI	91.9 a	11.1 c	49.3 b	56.7 b	38.3 ± 15.2	4.4–72.2
6 DAI	97.4 a	33.9 d	70.4 c	78.5 b	58.6 ± 17.7	12.2–88.9
7 DAI	100 a	62.2 d	84.2 c	91.9 b	74.9 ± 16.5	25.6–95.6
8 DAI	100 a	80.7 b	99.3 a	100 a	90.7 ± 11.6	51.1–100

^(x)^ DAI: days after incubation. Data are presented as mean germination rate or mean ± standard deviation. ^(y)^ Different letters indicate significant differences among lines at each DAI based on Tukey’s test (*p* < 0.05). ^(z)^ *n*: number of plants.

**Table 2:** List of QTLs identified for low-temperature germinability in F_2_ population.

Population	Trait ^(x)^	QTL	Chr.	Markers	*F*-Value	*p*-Value	R^2^ (%) ^(y)^	Gene Action ^(z)^		
								*a*	*d*	*d*/*a*
F_2_	LTG (6 DAI)	*qLTG1*	1	RM220-CRM22	14.4	0.000	16.0	10.0	0.2	0.0
		*qLTG3*	3	qLTG3-1_18D	23.1	0.000	23.8	9.6	9.7	1.0
		*qLTG10.2*	10	RM333-RM591	3.3	0.038	4.2	−4.2	5.1	−1.2
		Total					38.9			
	LTG (7 DAI)	*qLTG1*	1	RM220-CRM22	10.3	0.000	12.0	7.7	2.1	0.3
		*qLTG3*	3	qLTG3-1_18D	21.0	0.000	22.1	8.1	9.7	1.2
		*qLTG8*	8	RM72-RM22705	4.4	0.014	5.4	4.9	2.4	0.5
		*qLTG10.1*	10	RM25633	4.2	0.041	4.6	2.9	−1.9	−0.6
		*qLTG10.2*	10	RM333-RM591	5.4	0.006	6.6	−5.3	5.5	−1.0
		Total					42.0			

^(x)^ LTG: low-temperature germination. ^(y)^ R^2^: Coefficient of determination. ^(z)^ *a*: Additive effect = (*O. rufipogon* homozygote − Hwaseong homozygote)/2, *d*: Dominant effect = Heterozygote − (*O. rufipogon* homozygote + Hwaseong homozygote)/2, *d*/*a*: degree of dominance.

**Table 3:** Mean coleoptile lengths (mm) in *O. rufipogon*, Hwaseong, two introgression lines, and F_2_ population.

Coleoptile Lengths (mm)						
Trait ^(x)^	Lines (*n* = 5 ^(z)^)				F_2_ Population	
	*O. rufipogon*	Hwaseong	CR1517	CR1518	Mean	Range
7 DAI	2.6 a ^(y)^	0.7 c	1.6 b	1.1 bc	1.2 ± 0.7	0.2–9.2
8 DAI	4.5 a	1.4 d	2.8 b	2.0 c	3.2 ± 0.5	0.6–9.5
9 DAI	7.0 a	2.8 c	5.0 b	3.5 c	6.6 ± 1.3	2.0–10.0
10 DAI	9.0 a	3.8 d	7.4 b	5.6 c	7.9 ± 1.4	5.0–12.0

^(x)^ DAI: days after incubation. Data are presented as mean germination rate or mean ± standard deviation. ^(^^y)^ Different letters in each row indicate significant difference at *p* < 0.05 based on Tukey’s test. ^(z)^ *n*: number of plants.

**Table 4:** List of QTLs identified for coleoptile length under low-temperature condition.

Population	Trait ^(x)^	QTL	Chr.	Markers	*F*-Value	*p*-Value	R^2^ (%) ^(y)^	Gene Action ^(z)^		
								*a*	*d*	*d*/*a*
F_2_	CCL 9 DAI	*qCCL3*	3	qLTG3-1_18D	9.82	0.000	12.0	0.032	0.08	2.6
		*qCCL8*	8	RM22689-RM22705	4.77	0.001	6.2	0.043	0.02	0.6
	CCL 10 DAI	*qCCL1*	1	RM220-CRM22	4.97	0.008	6.4	0.037	0.02	0.1
		*qCCL3*	3	qLTG3-1_18D	7.30	0.001	9.2	0.033	0.07	2.3
		*qCCL8*	8	RM22689-RM22705	5.27	0.006	7.1	0.046	0.03	0.7

^(x)^ CCL: Coleoptile length, ^(y)^ Coefficient of determination, ^(z)^ *a*: Additive effect = (*O. rufipogon* homozygote-Hwaseong homozygote)/2, *d*: Dominant effect = Heterozygote − (*O. rufipogon* homozygote + Hwaseong homozygote)/2, *d*/*a*: Degree of dominance.

## Supplementary Materials

The following are available online at https://www.mdpi.com/2073-4425/11/10/1200/s1, Figure S1: Measurement of low-temperature germination rate of parental lines (*O. rufipogon*, Hwaseong, CR1517 and CR1518) for 4–8 days after incubation (DAI). Seeds were considered as germinated when coleoptile emerged. Arrows indicate emerged coleoptile, Figure S2: Comparison of germination rates of *O. rufipogon* (Ru), Hwaseong (Hs), CR1517 and CR1518 at normal (30 °C) (A) and low-temperature conditions (13 °C) (B). Different letters at each DAI indicate significant difference at *p* < 0.05 based on Tukey’s test. ns: no significant. Error bars indicate standard deviation, Figure S3: Interaction analysis of *qLTG1* and *qLTG3* with other QTL for LTG based on LTG scores of the F_2_ population. HH, HR and RR indicate Hwaseong homozygous, heterozygous (Hwaseong/*O. rufipogon*) and *O. rufipogon* homozygous at two QTLs, respectively. Low-temperature germination rates at 7 days after incubation were compared. Error bars indicate standard deviations. Interaction was determined by general linear regression model, Figure S4: Comparison of coleoptile length of parental lines at normal (30 °C) (A) and low-temperature (13 °C) (B) Error bars at each DAI indicate standard deviation. Different letters at each DAI indicate significant difference at *p* < 0.05 based on Tukey’s test. ns: no significant. Ru: *O. rufipogon*; Hs: Hwaseong, Table S1: List of primers used in this study, Table S2: Comparison of germination rates among parental lines and pyramiding lines at 6 and 7 DAI.
