# Supplementary material for: Characterization of Quantitative Trait Loci for Germination and Coleoptile Length under Low-Temperature Condition Using Introgression Lines Derived from an Interspecific Cross in Rice
Source: Genes (Basel). 2020 Oct 15;11(10):1200. doi: 10.3390/genes11101200 (PMC7650692; doi:10.3390/genes11101200)
Supplement: Supplementary file 1 [file genes-11-01200-s001.zip › genes-925906-suppl/supplimentary_figures_Submission.pptx]

## Slide 1
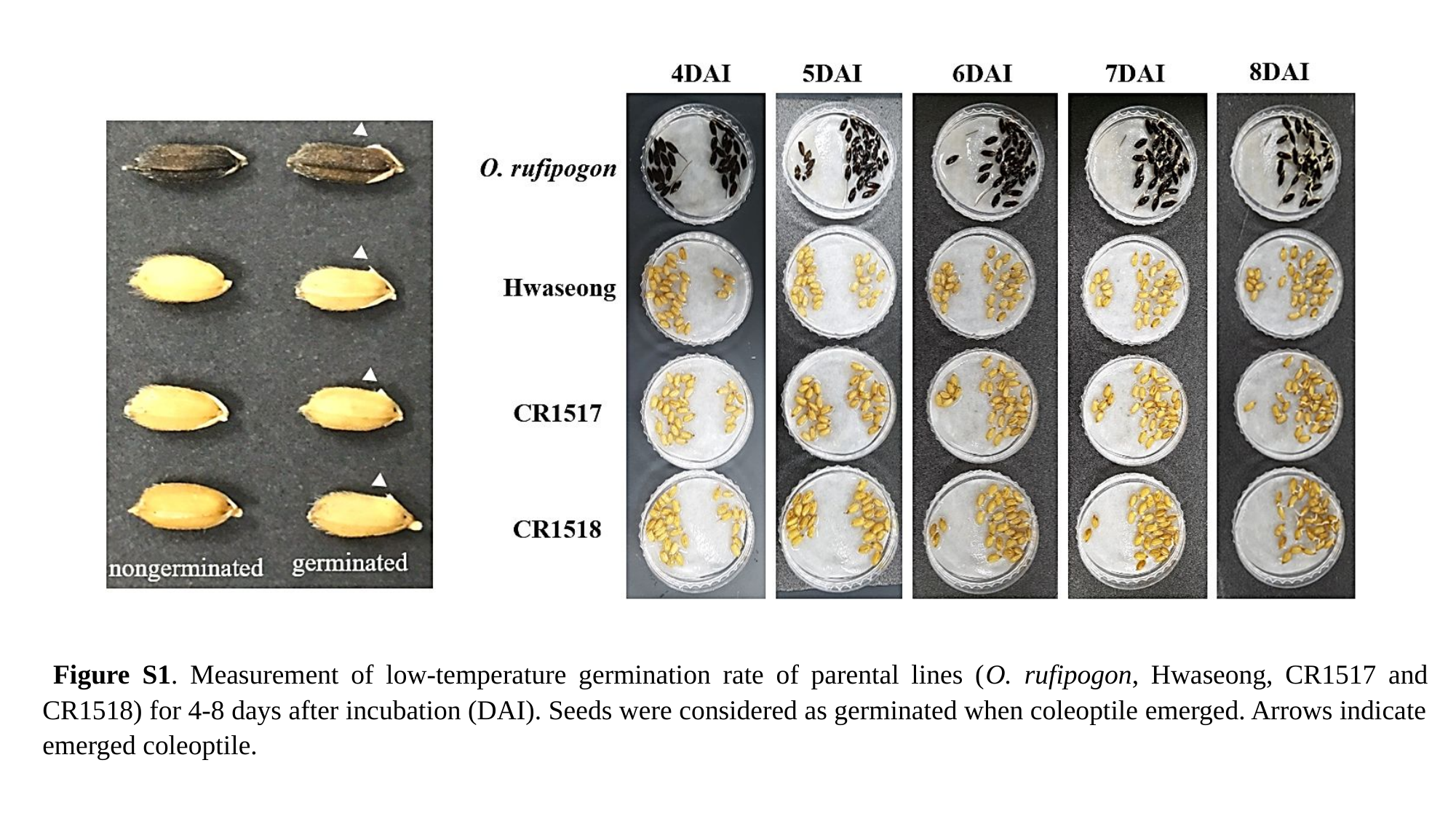

Figure S1. Measurement of low-temperature germination rate of parental lines (O. rufipogon, Hwaseong, CR1517 and CR1518) for 4-8 days after incubation (DAI). Seeds were considered as germinated when coleoptile emerged. Arrows indicate emerged coleoptile.

## Slide 2
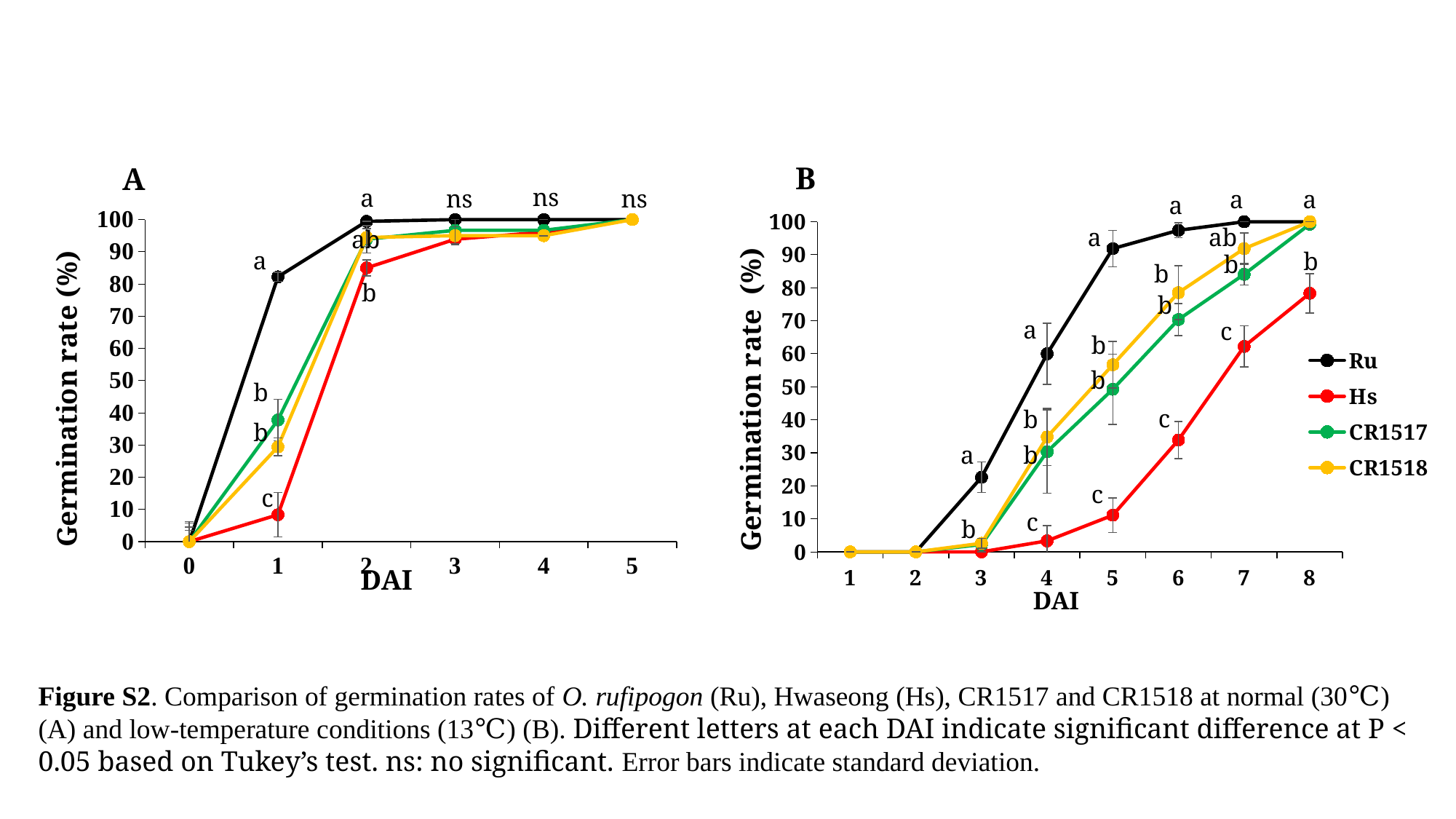

B
A
ns
a
ns
ns
a
a
a
### Chart
| Category | Ru. | HS | CR1517 | CR1518 |
|---|---|---|---|---|
| 0 | 0.0 | 0.0 | 0.0 | 0.0 |
| 1 | 82.22222222222223 | 8.333333333333332 | 37.777777777777786 | 29.444444444444446 |
| 2 | 99.44444444444443 | 85.0 | 93.88888888888889 | 94.44444444444444 |
| 3 | 100.0 | 93.88888888888889 | 96.66666666666667 | 95.0 |
| 4 | 100.0 | 96.1111111111111 | 96.66666666666667 | 95.0 |
| 5 | 100.0 | 100.0 | 100.0 | 100.0 |
### Chart
| Category | Ru | Hs | CR1517 | CR1518 |
|---|---|---|---|---|
| 1 | 0.0 | 0.0 | 0.0 | 0.0 |
| 2 | 0.0 | 0.0 | 0.0 | 0.0 |
| 3 | 22.59259259259259 | 0.0 | 2.222222222222222 | 2.5925925925925926 |
| 4 | 60.0 | 3.3333333333333335 | 30.37037037037037 | 34.81481481481482 |
| 5 | 91.85185185185186 | 11.111111111111112 | 49.25925925925927 | 56.666666666666664 |
| 6 | 97.4074074074074 | 33.8888888888889 | 70.37037037037037 | 78.51851851851852 |
| 7 | 100.0 | 62.22222222222222 | 84.07407407407406 | 91.85185185185186 |
| 8 | 100.0 | 78.33333333333334 | 99.25925925925925 | 100.0 |a
ab
ab
a
b
b
b
b
b
a
c
b
b
b
c
b
b
a
b
c
c
c
b
Figure S2. Comparison of germination rates of O. rufipogon (Ru), Hwaseong (Hs), CR1517 and CR1518 at normal (30℃) (A) and low-temperature conditions (13℃) (B). Different letters at each DAI indicate significant difference at P < 0.05 based on Tukey’s test. ns: no significant. Error bars indicate standard deviation.

## Slide 3
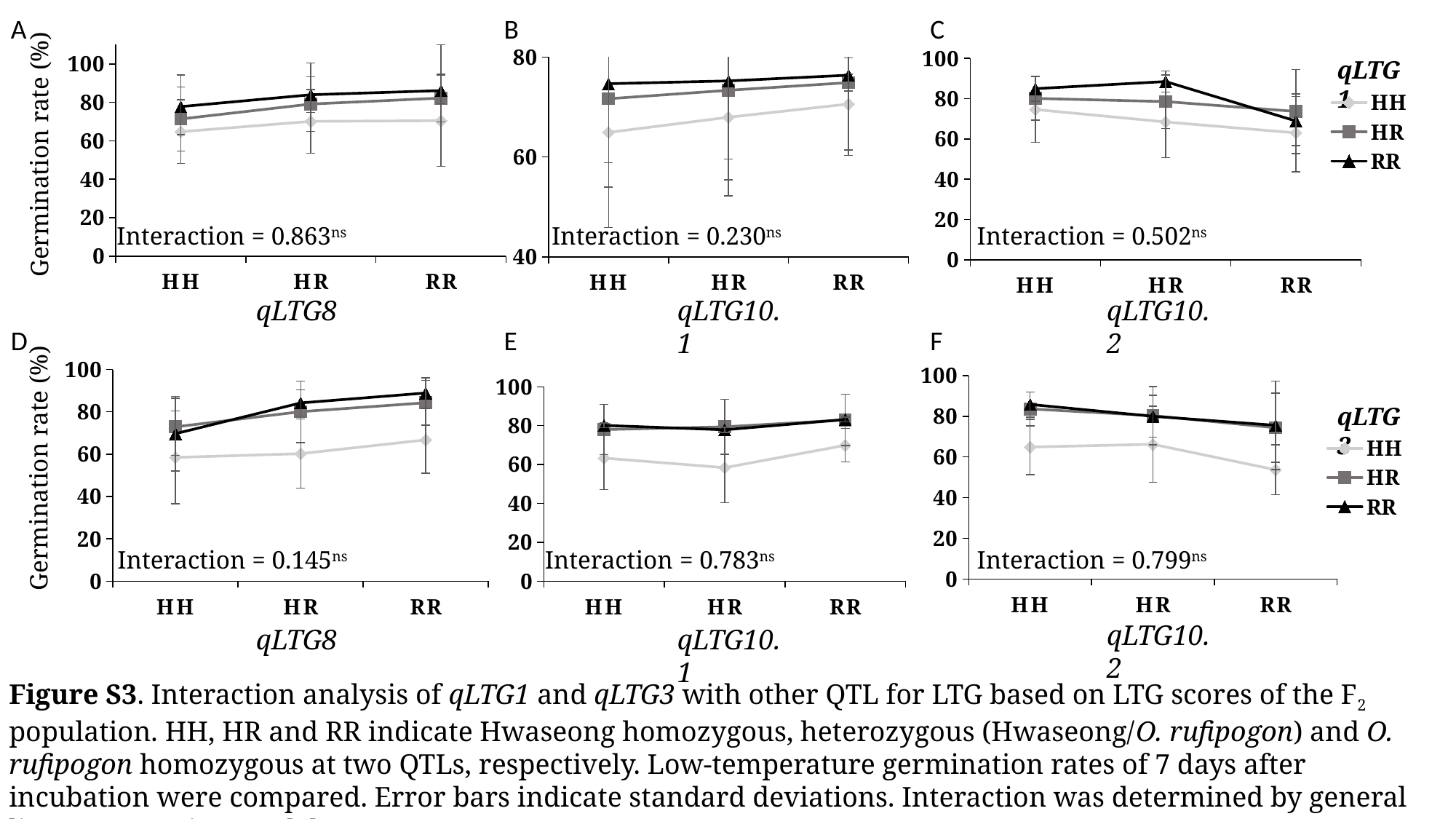

### Chart
| Category | HH | HR | RR |
|---|---|---|---|
| HH | 64.73 | 71.37 | 77.78 |
| HR | 70.1 | 79.11 | 83.94 |
| RR | 70.46 | 82.22 | 86.11 |A
B
C
### Chart
| Category | HH | HR | RR |
|---|---|---|---|
| HH | 74.57 | 80.07 | 84.92 |
| HR | 68.38 | 78.5 | 88.43 |
| RR | 62.98 | 73.61 | 68.89 |
### Chart
| Category | HH | HR | RR |
|---|---|---|---|
| HH | 64.92 | 71.66 | 74.67 |
| HR | 67.96 | 73.35 | 75.23 |
| RR | 70.6 | 74.889 | 76.37 |qLTG1
Germination rate (%)
Interaction = 0.863ns
Interaction = 0.230ns
Interaction = 0.502ns
qLTG8
qLTG10.1
qLTG10.2
D
E
F
### Chart
| Category | HH | HR | RR |
|---|---|---|---|
| HH | 64.89 | 83.64 | 85.83 |
| HR | 66.23 | 80.29 | 80.0 |
| RR | 53.68 | 74.38 | 75.56 |
### Chart
| Category | HH | HR | RR |
|---|---|---|---|
| HH | 58.47 | 72.93 | 69.7 |
| HR | 60.26 | 80.06 | 84.2 |
| RR | 66.77 | 84.29 | 88.89 |
### Chart
| Category | HH | HR | RR |
|---|---|---|---|
| HH | 63.33 | 77.99 | 80.12 |
| HR | 58.4 | 79.39 | 77.87 |
| RR | 69.89 | 82.96 | 83.17 |qLTG3
Germination rate (%)
Interaction = 0.145ns
Interaction = 0.783ns
Interaction = 0.799ns
qLTG10.2
qLTG8
qLTG10.1
Figure S3. Interaction analysis of qLTG1 and qLTG3 with other QTL for LTG based on LTG scores of the F2 population. HH, HR and RR indicate Hwaseong homozygous, heterozygous (Hwaseong/O. rufipogon) and O. rufipogon homozygous at two QTLs, respectively. Low-temperature germination rates of 7 days after incubation were compared. Error bars indicate standard deviations. Interaction was determined by general linear regression model.

## Slide 4
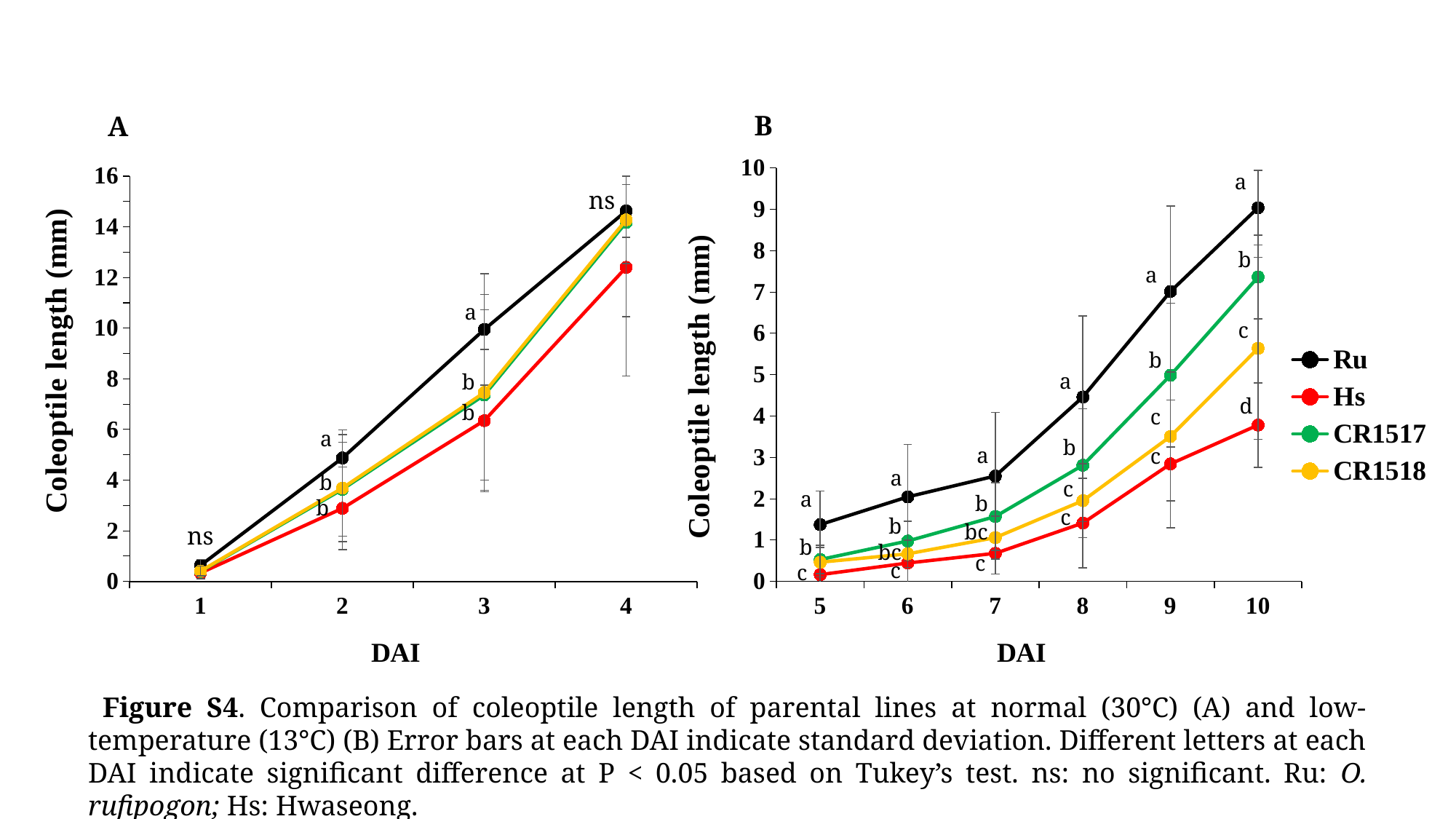

B
A
### Chart
| Category | Ru | Hs | CR1517 | CR1518 |
|---|---|---|---|---|
| 5 | 1.375 | 0.165 | 0.53 | 0.465 |
| 6 | 2.045 | 0.445 | 0.9775 | 0.667499999999999 |
| 7 | 2.55 | 0.68 | 1.57 | 1.06 |
| 8 | 4.4625 | 1.4125 | 2.81 | 1.9525 |
| 9 | 7.0125 | 2.8425 | 4.9875 | 3.5075 |
| 10 | 9.0375 | 3.7825 | 7.3625 | 5.6375 |
### Chart
| Category | Ru | Hs | CR1517 | CR1518 |
|---|---|---|---|---|
| 1 | 0.6375 | 0.3375 | 0.4 | 0.4125 |
| 2 | 4.875 | 2.8875 | 3.6375 | 3.6875 |
| 3 | 9.95 | 6.35 | 7.3625 | 7.4625 |
| 4 | 14.625 | 12.4 | 14.175 | 14.275 |a
ns
b
a
a
c
b
a
b
d
b
c
a
b
a
c
a
b
c
a
b
b
c
b
bc
ns
b
bc
c
c
c
 Figure S4. Comparison of coleoptile length of parental lines at normal (30°C) (A) and low-temperature (13°C) (B) Error bars at each DAI indicate standard deviation. Different letters at each DAI indicate significant difference at P < 0.05 based on Tukey’s test. ns: no significant. Ru: O. rufipogon; Hs: Hwaseong.
